# Clinical and Cardiovascular Profile in Patients With Peripheral Artery Disease

**DOI:** 10.7759/cureus.39586

**Published:** 2023-05-28

**Authors:** Fawaz Bardooli, Rani Al Agha, Dileep Kumar

**Affiliations:** 1 Interventional Cardiology, Mohammed Bin Khalifa Specialist Cardiac Centre, Awali, BHR; 2 Vascular Surgery, Al Salmaniya Hospital, Manama, BHR; 3 Cardiology, Mohammed Bin Khalifa Specialist Cardiac Centre, Awali, BHR

**Keywords:** " "cardiovascular disease risk factors, myocardial infarction, carotid atherosclerosis, coronary artery lesion (cal), peripheral arterial diseases

## Abstract

Background

Peripheral artery disease (PAD) is a vascular disorder leading to serious complications if not managed promptly. This study is conducted to analyze clinical and cardiovascular risk factors in PAD patients presenting at a tertiary care hospital and management strategies.

Methodology

This observational study was conducted at the Department of Cardiology, Mohamed Bin Khalifa Specialist Cardiac Centre. One hundred and twenty patients aged more than 35 years with PAD were included in the study. Data regarding age, gender, physical exam, cardiovascular risk profile, carotid disease, coronary artery disease, and treatment strategy were recorded on a pre-designed questionnaire by the researcher himself. The data were analyzed using IBM Corp. Released 2017. IBM SPSS Statistics for Windows, Version 25.0. Armonk, NY: IBM Corp.

Results

The mean age of patients with PAD was 65.46±10.56 years. About 79.2% were hypertensive, 81.7% had hyperlipidemia, 83.3% had diabetes, 29.2% had renal insufficiency, and 38.3% were active smokers, respectively. In age ≥65 years, infra-popliteal PAD was significantly lower as compared to above-knee PAD (23.4% vs. 76.6%, p=0.002). In diabetic patients, the proportion of above-knee PAD was higher than below-knee PAD (60% vs. 40%, p=0.033).

Conclusion

Older age, diabetes, and carotid disease were significant predictors for peripheral artery disease, and these are significantly associated with above-the-knee peripheral artery disease.

## Introduction

Peripheral artery disease (PAD) is a leading cause of morbidity [1]. This is a serious vascular condition characterized by debilitating atherosclerotic occlusion of lower limb arteries [2]. This is typically associated with multi-vessel disease, which raises the risk of morbidity and mortality and affects up to 14% of the general population [3,4]. In South Asia, there were 1,286,587 new cases of PAD, and 2964 deaths occurred due to it. Moreover, there were 68404 new cases of PAD among females and 43,876 new cases of PAD among males [5].

Higher age, diabetes, smoking, hypertension, hyperlipidemia, male gender, raised glucose levels, raised plasma fibrinogen levels, heart failure, previous history of myocardial infarction, and cerebrovascular events are the factors associated with PAD [1,6-8]. A Pakistani study revealed that PAD was significantly associated with smoking (p=0.001), obesity (p=0.004), hypertension (p=0.001), and hypercholesterolemia (p=0.005) [9].

If PAD is left untreated, it raises the risk of stroke, heart attack, amputation, and mortality. [6] As atherosclerosis interrupts several circulatory subsystems, there is a greater risk of cerebrovascular disease and coronary artery disease among patients with PAD [6,10]. Various pieces of research have revealed that myocardial infarction patients with PAD are at higher risk of adverse cardiac events than myocardial infarction patients without PAD [11-13]. Another research revealed that patients with PAD had a four-fold greater risk of overall mortality and an approximately eight-fold greater risk of cardiovascular death. Furthermore, PAD is significantly associated with disability and poor quality of life [14].

In the Gulf region, data on the clinical and cardiovascular profiles of individuals with PAD are scarce. Better clinical and cardiovascular profile information would improve the treatment outcomes of individuals with atherosclerotic cardiovascular disease in clinical practice. As a result, the current study sought to analyze clinical and cardiovascular risk factors and the management of PAD patients presenting at a tertiary care hospital.

## Materials and methods

This was an observational study conducted at the Department of Cardiology at the Mohamed Bin Khalifa Specialist Cardiac Centre in Bahrain. The sample size of 120 patients with PAD was estimated using the Open Epi sample size calculator by taking the statistics of heart disease among PAD as 34% [15], the bond on an error as 8.5%, and the 95% confidence level. Patients aged more than 35 years of either gender with PAD were included in the study. PAD was labeled as positive if patients had a pathological ankle-brachial index (ABI) less than 0.90 in at least one lower extremity or a history of amputation or revascularization because of ischemia. Patients with indications of anticoagulation or a history of atrial fibrillation were excluded from the study. Patients for the present study were selected using the consecutive sampling technique.

The ethical committee of NICVD approved this study, and verbal informed consent was obtained from all the eligible participants before enrollment. Data regarding age, gender, physical exam (weight, height, BMI), cardiovascular risk profile (smoking, diabetes, hypertension, hyperlipidemia, and renal insufficiency), carotid disease, coronary artery disease (acute myocardial infarction [MI], coronary artery bypass graft surgery [CABG], and percutaneous coronary intervention [PCI]), and treatment strategy (pre-dilatation and provisional stenting) were recorded on a pre-designed questionnaire by the researcher himself.

The data were analyzed using IBM Corp. Released 2017. IBM SPSS Statistics for Windows, Version 25.0. Armonk, NY: IBM Corp. The mean and standard deviation were reported for age and BMI. Frequency and percentage were computed for gender, cardiovascular risk profile, peripheral artery disease, and carotid disease, coronary artery disease (PCI, CABG, and MI). Comparisons between peripheral artery disease (above and below the knee) and carotid disease, coronary artery disease (CAD), and types of CAD such as PCI, CABG, and MI were done using the chi-square test/Fisher exact test. A p-value of ≤0.05 was considered statistically significant.

## Results

The mean age and BMI of the included patients were 65.46±10.56 years and 28.68±5.45 kg/m2. Of 120 patients, most were males (85.8%), and 14.2% were females. About 79.2% were hypertensive, 81.7% had hyperlipidemia, 83.3% had diabetes, 29.2% had renal insufficiency, and 38.3% were smokers (Table 1). 

**Table 1:** Baseline characteristics of study variables

Variables	Statistics
Age (years)	65.46±10.56
BMI (kg/m2)	28.68±5.45
Gender	
Male	103 (85.8%)
Female	7 (14.2%)
Comorbidities	
Hypertension	95 (79.2%)
Hyperlipidemia	98 (81.7%)
Diabetes	100 (83.3%)
Renal insufficiency	35 (29.2%)
Active smoking	46 (38.35)

A statistically significant difference was observed between PAD and carotid disease (p=0.028). While an insignificant difference was observed between overall CAD and categories of CAD with respect to PAD (p>0.05) (Table 2).

**Table 2:** Cardiovascular profile PAD: Peripheral artery disease

PAD	Carotid disease	Coronary artery disease (n=67, 55.8%)		
		PCI	CABG	Acute MI
Below knee (n=43, 35.8%)	0	19 (43.2%)	7 (33.3%)	1 (50%)
Above knee (n=77, 64.2%)	8 (100%)	25 (56.8%)	14 (66.7%)	1 (50%)
Total (n=120)	8	44	21	2
p-value	0.028*	0.238	0.81	0.999
		0.251		

Of 120 patients, predilatation (only ballooning) was performed in 109 patients (90.8%), and stenting after dilatation was performed in 24 patients (20%). A statistically insignificant difference was observed between management strategy and PAD with a p-value >0.05 (Table 3).

**Table 3:** Comparing treatment methods PAD: Peripheral artery disease

Treatment method	PAD		p-value
	Below knee (n=43, 35.8%)	Above knee (n=77, 64.2%)	
Predilatation (n=109)	37 (33.9%)	72 (66.1%)	0.174
Stenting (n=24)	9 (37.5%)	15 (62.5%)	0.849

In age ≥65 years, below-knee PAD was significantly lower as compared to above-knee PAD (23.4% vs. 76.6%, p=0.002). The odds of above-knee PAD were 3.26 times higher in patients of age ≥65 years than those aged <65 years. In diabetic patients, the proportion of above-knee PAD was higher than below-knee PAD (60% vs. 40%, p=0.033). The odds of above-knee PAD were 3.77 times higher among diabetic patients as compared to non-diabetic patients (Table 4). 

**Table 4:** Peripheral artery disease and cardiovascular risk factors PAD: Peripheral artery disease

	PAD		p-value	OR (95% CI)	
Age groups	Below knee	Above knee			
<65 years	28 (50%)	28 (50%)	0.002*	3.26 (1.49-7.12)	
≥65 years	15 (23.4%)	49 (76.6%)			
BMI					
<30 kg/m2	29 (38.2%)	47 (61.8%)	0.485	1.32 (0.60-2.90)	
≥30 kg/m2	14 (31.8%)	30 (68.2%)			
Gender					
Male	35 (34%)	68 (66%)	0.297	0.57 (0.20-1.63)	
Female	8 (47.1%)	9 (52.9%)			
Hypertension					
Yes	36 (37.9%)	59 (62.1%)	0.359	1.56 (0.59-4.12)	
No	7 (28%)	18 (72%)			
Hyperlipidemia					
Yes	35 (35.7%)	63 (64.3%)	0.954	0.97 (0.37-2.54)	
No	8 (36.4%)	14 (63.6%)			
Diabetes					
Yes	40 (40%)	60 (60%)	0.033*	3.77 (1.03-13.73)	
No	3 (15%)	17 (85%)			
Smoking					
Yes	19 (41.3%)	27 (58.7%)	0.324	1.46 (0.68-3.14)	
No	24 (32.4%)	50 (67.6%)			
Renal insufficiency					
Yes	11 (31.4%)	24 (68.6%)	0.518	0.75 (0.32-1.75)	
No	32 (37.6%)	53 (62.4%)			

## Discussion

The prevalence of PAD in the general population is 14% among older people [3,4]. In Asian-Pacific countries, the prevalence rates of PAD range from 5% to 12.1%. For diabetic patients with PAD, the prevalence is reported as 19.4% in Chinese, 6% in Malays, 20% in Indians, and 32% in Pakistanis [16,17]. The variation in geography, socioeconomic status, and lifestyle determinants might play a role in the differences in PAD among different populations [18,19].

Most of the patients in our study were aged equal to 65 years, and the mean age was 65.46±10.56 years. Studies also revealed that the risk of PAD increases with age. Wu et al. showed that the mean age of patients with PAD associated with below-knee amputation was 66 years, ranging from 41 to 97 years [20]. Literature shows that the proportion of PAD is approximately 5% in the age group 40 to 44 years and approximately 12% in 70 to 74 years in males and females in developed countries [21,22]. In developing countries, the proportion of PAD among females is almost the same as in developed countries. Moreover, in developing countries, PAD is estimated at 2% to 8% among males [21,22]. 

The known risk factors identified for below-knee PAD in the current study were age, diabetes, and carotid disease. Research studies have revealed that male gender, older age, hyperlipidemia, smoking, diabetes, hypertension, acute MI, and stroke are the predictors of PAD [1,6-8]. Attar et al. found that in Swedish PAD patients with MI, the proportion of PAD was higher in patients with previous MI, hypertension, diabetes, and older age [13]. Escobar et al. also found that PAD was frequent among diabetic patients older than 70 years [1]. Umer et al. in Pakistan found that higher age, hypertension, a longer duration of diabetes, and smoking were significant predictors for PAD. Another Pakistani study by Majid et al. revealed that PAD was significantly associated with smoking (p=0.001), obesity (p=0.004), hypertension (p=0.001), and hypercholesterolemia (p=0.005) [9]. Urbano et al. found that hypertension was the most significant risk factor for PAD, with odds of 4.9 times, followed by diabetes mellitus, dyslipidemia, and a higher BMI [23].

In the current study, we found that the proportion of coronary artery diseases was statistically similar between below- and above-knee PAD. Different studies have revealed that MI patients with PAD have a greater risk of cardiovascular events as compared to MI patients without PAD [11-13]. Previous studies also revealed that the risk of overall mortality and cardiovascular mortality is higher among patients with PAD [14].

Hence, our study highlighted the importance of risk profile assessment in high-risk populations to screen out people at high risk. This step would help in early intervention and follow-up of high-risk populations to improve treatment strategies for decreasing the burden of cardiovascular diseases. More prospective trials with a larger sample size should be conducted to increase the generalizability of findings.

## Conclusions

Older age, diabetes, and carotid disease were significant predictors of peripheral artery disease, and these are remarkably associated with above-the-knee femoropopliteal peripheral artery disease. Prompt diagnosis and management are crucial to preventing serious complications. Screening for other vascular disorders may be considered for PAD patients.

## References

[1] Escobar C, Blanes I, Ruiz A (2011). Prevalence and clinical profile and management of peripheral arterial disease in elderly patients with diabetes. Eur J Intern Med.

[2] Cimminiello C, Borghi C, Kownator S (2010). Prevalence of peripheral arterial disease in patients at non-high cardiovascular risk. Rationale and design of the PANDORA study. BMC Cardiovasc Disord.

[3] Farag S, Elbalkimy M, Elbassiouny A, George J, Fathy M (2020). Prevalence of peripheral arterial diseases in patients with large artery ischemic stroke and its prognostic value. Egyp Jr Neur Psyc Neur.

[4] Aursulesei Onofrei V, Ceasovschih A, Marcu DT, Adam CA, Mitu O, Mitu F (2022). Mortality risk assessment in peripheral arterial disease-the burden of cardiovascular risk factors over the years: a single center's experience. Diagnostics (Basel).

[5] Lin J, Chen Y, Jiang N, Li Z, Xu S (2022). Burden of peripheral artery disease and its attributable risk factors in 204 countries and territories from 1990 to 2019. Front Cardiovasc Med.

[6] Zanati SG, Mouraria GG, Matsubara LS, Giannini M, Matsubara BB (2009). Profile of cardiovascular risk factors and mortality in patients with symptomatic peripheral arterial disease. Clinics (Sao Paulo).

[7] Barrios V, Escobar C, Suarez C, Garcia-Moll X, Lozano F (2022). Clinical profile and management of patient patients with ischemic heart disease and/or peripheral artery disease in clinical practice: the APALUSA study. J Clin Med.

[8] Paquissi FC, Cuvinje AB, Cuvinje AB (2016). Prevalence of peripheral arterial disease among adult patients attending outpatient clinic at a general hospital in South Angola. Scientifica (Cairo).

[9] Majid Khan A, Lohana P, Anvekar P (2021). Risk factors of peripheral vascular disease in diabetes mellitus in Abbottabad, Pakistan: a cross-sectional study. Cureus.

[10] Shah AM, Banerjee T, Mukherjee D (2008). Coronary, peripheral and cerebrovascular disease: a complex relationship. Herz.

[11] Grenon SM, Vittinghoff E, Owens CD, Conte MS, Whooley M, Cohen BE (2013). Peripheral artery disease and risk of cardiovascular events in patients with coronary artery disease: insights from the Heart and Soul Study. Vasc Med.

[12] Inglis SC, Bebchuk J, Al-Suhaim SA (2013). Peripheral artery disease and outcomes after myocardial infarction: an individual-patient meta-analysis of 28,771 patients in CAPRICORN, EPEHESUS, OPTIMAAL and VALIANT. Int J Cardiol.

[13] Attar R, Wester A, Koul S, Eggert S, Andell P (2019). Peripheral artery disease and outcomes in patients with acute myocardial infarction. Open Heart.

[14] Brevetti G, Schiano V, Verdoliva S (2006). Peripheral arterial disease and cardiovascular risk in Italy. Results of the Peripheral Arteriopathy and Cardiovascular Events (PACE) study. J Cardiovasc Med (Hagerstown).

[15] Stoia MA, Farcaș AD, Anton FP, Roman AI, Vida-Simiti LA (2016). Comparative analysis of cardiovascular risk profile, cardiac and cervical arterial ultrasound in patients with chronic coronary and peripheral arterial ischemia. Inter Conf Adv Med Hea Car Tech.

[16] Abola MT, Golledge J, Miyata T (2020). Asia-Pacific consensus statement on the management of peripheral artery disease: A report from the Asian Pacific Society of Atherosclerosis and Vascular Disease Asia-Pacific Peripheral Artery Disease Consensus Statement Project Committee. J Atheroscler Thromb.

[17] Akram J, Aamir AU, Basit A (2011). Prevalence of peripheral arterial disease in type 2 diabetics in Pakistan. J Pak Med Assoc.

[18] Demsas F, Joiner MM, Telma K, Flores AM, Teklu S, Ross EG (2022). Disparities in peripheral artery disease care: A review and call for action. Semin Vasc Surg.

[19] Vart P, Coresh J, Kwak L, Ballew SH, Heiss G, Matsushita K (2017). Socioeconomic status and incidence of hospitalization with lower-extremity peripheral artery disease: atherosclerosis risk in communities study. J Am Heart Assoc.

[20] Wu JT, Wong M, Lo ZJ, Wong WE, Narayanan S, Tan GW, Chandrasekar S (2017). A series of 210 peripheral arterial disease below-knee amputations and predictors for subsequent above-knee amputations. Ann Vasc Dis.

[21] Criqui MH, Matsushita K, Aboyans V (2021). Lower extremity peripheral artery disease: contemporary epidemiology, management gaps, and future directions: a scientific statement from the American Heart Association. Circulation.

[22] Fowkes FGR, Rudan D, Rudan I (2013). Comparison of global estimates of prevalence and risk factors for peripheral artery disease in 2000 and 2010: a systematic review and analysis. Lancet.

[23] Urbano L, Portilla E, Muñoz W, Hofman A, Sierra-Torres CH (2018). Prevalence and risk factors associated with peripheral arterial disease in an adult population from Colombia. Arch Cardiol Mex.

